# Sex-dependent modulation of ultrasonic vocalizations in house mice (*Mus musculus musculus*)

**DOI:** 10.1371/journal.pone.0188647

**Published:** 2017-12-13

**Authors:** Sarah M. Zala, Doris Reitschmidt, Anton Noll, Peter Balazs, Dustin J. Penn

**Affiliations:** 1 Department of Integrative Biology and Evolution, Konrad Lorenz Institute of Ethology, University of Veterinary Medicine, Vienna, Austria; 2 Acoustic Research Institute, Austrian Academy of Sciences, Vienna, Austria; University of Missouri Columbia, UNITED STATES

## Abstract

House mice (*Mus musculus)* emit ultrasonic vocalizations (USVs), which are surprisingly complex and have features of bird song, but their functions are not well understood. Previous studies have reported mixed evidence on whether there are sex differences in USV emission, though vocalization rate or other features may depend upon whether potential receivers are of the same or opposite sex. We recorded the USVs of wild-derived adult house mice (F1 of wild-caught *Mus musculus musculus*), and we compared the vocalizations of males and females in response to a stimulus mouse of the same- or opposite-sex. To detect and quantify vocalizations, we used an algorithm that automatically detects USVs (*Automatic Mouse Ultrasound Detector* or *A-MUD*). We found high individual variation in USV emission rates (4 to 2083 elements/10 min trial) and a skewed distribution, with most mice (60%) emitting few (≤50) elements. We found no differences in the rates of calling between the sexes overall, but mice of both sexes emitted vocalizations at a higher rate and higher frequencies during opposite- compared to same-sex interactions. We also observed a trend toward higher amplitudes by males when presented with a male compared to a female stimulus. Our results suggest that mice modulate the rate and frequency of vocalizations depending upon the sex of potential receivers.

## Introduction

House mice (*Mus musculus)* produce surprisingly complex ultrasonic vocalizations (USVs) during social and sexual interactions, which have features similar to bird song [1, 2]. Mice are an intensively studied species, and their USVs have great potential to provide a model system for basic research on animal communication in behavioral biology and neuroscience, and also applied questions in biomedical sciences (see reviews [2, 3, 4–7]). Mouse USVs appear to provide a variety of functions, including social recognition (individual, kin, sex, and species recognition), intimidating rivals (intra-sexual selection), and attracting mates (inter-sexual selection) (see reviews [8, 9]). USVs consist of several types of calls with distinctive temporal-spectral characteristics (> 10 types of ‘syllables’ have been classified) [7, 10, 11], and they are often emitted in bouts (‘phrases’) of repeated sequences [1]. USVs appear to be largely ‘innate’ because syllable repertoires, which differ among inbred mouse strains [10, 12], are not altered by cross-fostering [13]. Moreover, auditory feedback is not required to produce apparently normal vocalizations [2, 14, 15]. Yet, mice modulate many features of USVs, including frequency, duration, amplitude, syntax, and especially vocalization rate, which are influenced by a variety of factors, including age, sex, genetic background, and behavioral/physiological state (reviewed in [7]) and social context (laboratory mice [11, 16, 17]; wild-derived mice: [18]). Our aim in this study was to investigate sex differences in the vocalizations of wild house mice (*Mus musculus musculus*), as a step towards determining the functions of these complex signals (see reviews [8, 9]).

USVs have been mainly studied in classical inbred strains of laboratory mice, *Mus laboratorius* [19], which are mostly hybrids of *Mus* species and subspecies [20] and there are still few studies on wild house mice. Although domesticated laboratory mice provide useful models for USV studies, it is unclear whether the findings generalize to wild mice [21] or even to other laboratory strains [10, 13, 22]. There have been few studies on the vocalizations of wild house mice, and previous studies on the *Mus musculus musculus* subspecies were conducted only on male USVs elicited by urinary odors, and female responses to male USV playbacks [8, 21, 23–26]. Comparing the USVs of the sexes–and determining the contexts in which wild mice vocalize and how they modulate their vocalizations–should help provide insights into their functions. Results from previous studies on sex differences in mouse USVs are inconsistent. Sex differences in USV emission are not as great as once thought, but some differences may have been overlooked in recent studies [7]. Therefore, our aims here were to compare the vocalizations of males and females during encounters with individuals of the same and opposite sex in wild-derived house mice.

Most USV studies have focused on male mice, and they have generally found that males emit USVs at particularly high rates in response to female stimuli, as part of their courtship behavior (see reviews [8, 9]). Males emit USVs upon encountering the scent of adult females (particularly fresh urine (wild-derived mice: [23]; laboratory mice: [11])), whereas they emit few if any calls for the scent of immature females or males (wild-derived mice: [24]; laboratory mice: [11, 27–29]). During direct interactions with females, laboratory males vocalize particularly while chasing [30], anogenital sniffing, and copulation [10, 29, 31–34], and they alter their USVs when interacting with females in estrous compared to unreceptive females [31–33]. Laboratory males produce mainly short syllables upon initial interactions with receptive females, which become more complex during courtship and mounting [29, 35], and then abruptly cease after ejaculation [29, 31, 36, 37]. Females are attracted to vocalizing over non-vocalizing (surgically muted) males (laboratory mice: [38]), to recorded playbacks of male USVs (wild-derived mice: [24]; laboratory mice: [12, 39, 40]), and to males producing more complex USVs (laboratory mice: [11]). In wild-derived mice, females’ attraction towards male USVs potentially facilitates mate choice for male quality and genetic compatibility (i.e., avoiding inbreeding [21, 24] and heterospecific matings [26]). Thus, previous studies suggest that male mice modulate USV emission depending upon the sex (and estrous status) of potential receivers. Hence, USVs appear to be a secondary sexual trait, which potentially evolves under sexual selection, as with scent marking and chemical signals [41]. Although male mice do not emit USVs in response to male odor, males emit USVs during direct same-sex interactions, which may mediate competition over social status (intra-sexual selection) (laboratory mice: [42, 43, 44]). However, the functions of male USVs emitted during same-sex encounters are not understood, and they have received surprisingly little attention.

Female house mice also emit USVs, however, the contexts and functions of their vocalizations are not well understood. Early studies concluded that females call at lower rates than males, and mainly or exclusively during same-sex interactions. Female laboratory mice were found to emit USVs during direct female-female interactions [45], and in response to anesthetized females [46], though not to female odor [46, 47]. Females were also found to vocalize at low rates during direct female-female encounters (35± 22 calls/min) compared to males calling for female odor (144±43 calls/min) [47]. For example, during resident-intruder tests, females vocalized to females (unless separated by a perforated and transparent partition) and USVs appeared to be emitted mainly by resident females (i.e., resident females vocalized to anaesthetized intruders, whereas intruders did not vocalize to an anaesthetized resident) [47]. Unlike males, females were found to emit few if any USVs in response to the odor of the opposite sex [46] or during direct male-female interactions [37, 48, 49].

Three more recent studies, however, examined female vocalizations and their results are inconsistent with the above findings, though only one of these studies recorded both sexes during same- and opposite-sex interactions. First, Hammerschmidt et al. (2012) compared the USVs of the sexes following the introduction of an unfamiliar mouse, either awake or anesthetized, into a resident’s cage (3-min recordings of C57BL/6NCrl strain) [50]. As expected, resident males emitted more USVs when confronted with a female compared to a male intruder, but surprisingly, females vocalized at much (>3x) higher rates than males upon encountering a female intruder. Females’ responses to male intruders were not tested, however, which is necessary to compare same- versus opposite-sex interactions. Only minor sex differences in call structure were detected, but since only three types of calls were identified, analyses with finer resolution are needed [7]. Second, Von Merten et al. (2014) recorded wild-derived mice (F3 to F5; *Mus musculus domesticus*) over two days, and compared the USV emission of both sexes during same- and opposite-sex interactions, while separated by a clear perforated partition [51]. Sex differences in USVs were found, and females emitted similar rates of vocalizations than males during opposite-sex encounters, whereas females vocalized at higher rates than males during same-sex encounters. Third, Neuenuebel et al. (2015) recorded groups of laboratory mice using a microphone array that allowed vocalizations to be assigned to individuals, and showed that both sexes emit USVs when directly interacting [30]. This study suggests the intriguing possibility that courtship USV emission is an interactive process between the sexes, however, it did not rule out the possibility that females vocalized in response to the other females in the group, which may have provided a direct stimulus or an audience effect [52].

In summary, there is mixed evidence regarding sex differences in USV rate, though USV emission may depend upon the sex of the stimulus (target receivers), and there may be interactions. It is also unclear how well previous results from classical inbred strains on these questions extend to wild and wild-derived mice. Therefore, we aimed to compare the USVs of sexes, and determine whether one or both sexes modulates calling in responses to same- versus opposite-sex individuals. We recorded wild-derived house mice (F1 offspring of wild-caught *Mus musculus musculus*) during the presentation of an individual stimulus of the same or opposite sex. The stimulus animal was housed in a separate covered compartment, which enabled us to record the vocalizations of the subject, and not the stimulus mouse. We aimed to test whether males or females emit more USVs upon encountering a stimulus individual (sexual dimorphic emission), and whether vocalizations of either sex depend upon the sex of the stimulus receiver (sex-specific modulation). We expected high individual variation in USV emission, and that some individuals would produce few, if any, USVs, given previous results with wild-derived mice [21, 24, 26, 51]. We also expected that males would vocalize at higher rates during opposite- than same-sex interactions, compared to when presented with odor samples [24]. We had no clear predictions about which sex would vocalize at higher rates, given the conflicting results of previous studies, though we expected that mice of both sexes might modulate their calling depending upon the sex of the stimulus individual, as suggested by previous studies.

## Materials and methods

### Subjects and housing

Our study was conducted with wild-derived house mice (*Mus musculus musculus*), which were the F1 offspring of 60 wild house mice caught at seven locations of the *Konrad Lorenz Institute of Ethology* (48°12’38”N, 16°16’54”E) campus in Vienna, Austria. The locations were separated by buildings, aviaries, other animal facilities, paths and woods (mean±SD of the distance between locations was 84 ± 71 m). The wild-caught mice were systematically crossed between locations (to form 30 breeding pairs) and mice were never crossed from the same location to reduce the risk of close inbreeding. We did not study wild-caught mice because we aimed to control for variation in age, rearing conditions and other differences, such as close relatedness or inbreeding. The F1 offspring were housed in mixed-sex family groups (standard Type IIL cages, 36.5 x 20 x 14 cm, with stainless steel cover, 1cm mesh width, Tecniplast, Germany) until weaning (21 d of age). After weaning, siblings were housed in mixed-sex groups with a maximum of four mice per cage, and at 5 weeks of age, the sexes were separated. Females were housed in sister pairs and males were individually housed to prevent fighting. All cages were provided equally with nesting material (Nestlet, Ehret, Austria), wood shavings (ABEDD, Austria), one cardboard paper roll and one nest box (Tecniplast, Germany) for environmental enrichment. Water and food (rodent diet 1324, Altromin, Germany) were provided *ad libitum*. Mice were kept in standard conditions (mean±SD room temperate: 22 ± 2°C, in a 12:12 h light:dark cycle, lights off at 15:00). Red light was used instead of a complete dark period to be able to conduct experiments during the active period for mice without disturbing them. We used 80 adult mice (n = 40 males, n = 40 females, mean ± SD age: 259 ± 23d).

### Recording apparatus and general procedure

We recorded the vocalizations of individual focal subjects (“callers”) in the presence of an unfamiliar “stimulus” mouse, which was haphazardly chosen from another family and thus was not closely related (not a first-degree relative) to the caller. We recorded under red light, during the active period of the day for mice (15:00 to 17:30). All callers were socially experienced (“primed”), as we placed an unfamiliar, not closely related mouse into the subject’s home cage (for 5 min) 1 d prior to the recordings. Stimulus mice were used once as priming animals and once as stimulus, but never for the same calling subject. Thus, all callers were always unfamiliar and not closely related to the stimuli and priming animals. We used a small plastic cylinder to gently transfer the mice from their cages into the experimental compartments of a Plexiglas cage (36.5 x 21 x 15 cm). To ensure that the mice could see and smell each other during the experiment the two compartments were separated by a 0.5 cm thick Plexiglass divider covered with small holes (0.5 cm diameter). The ‘caller compartment’ was covered with a metal cage lid (1 cm width mesh), whereas the ‘stimulus compartment’ was covered with a Plexiglass lid to prevent recording USVs from the stimulus animal. We used USV playbacks from an ultrasound speaker (Avisoft Bioacoustics, Germany) positioned into the stimulus compartment, to confirm that the Plexiglass cover was very effective at blocking USVs. The stimulus compartment was also provided with bedding and 2–3 food pellets.

To record, we first placed the stimulus mouse into the assigned compartment and after 5–10 min habituation time, we introduced the focal mouse. The entire cage was then positioned inside a recording chamber, which was lined with acoustic foam as described in [26]. A condenser ultrasound microphone (Avisoft Bioacoustics/CM16/CMPA with an integrated pre-amplifier and a frequency range from 10 to 200 kHz) and an UltraSoundGate 116–200 (Avisoft Bioacoustics, Germany) were mounted inside the recording chamber, 10 cm above the caller compartment. Before each recording, the microphone was calibrated with a 440 Hz tone of a commercial available tuning fork. Mice were recorded using the RECORDER USGH software with settings at 300 kHz sampling rate, 16 bit format, and 256 Hz FFT size. After positioning the cage inside the recording chamber, we waited for 30 sec and then started recording for 10 min. To standardize any potential estrus status effects of stimulus females, we included an olfactory stimulus (5 μl of urine pooled from 4 different females pipetted onto 4 x 4 cm filter paper) into the caller compartment. The urine was collected in metabolic cages (Techniplast, 600M021) from wild-caught adult females, equally aliquoted in Eppendorf tubes and stored at -20°C until the recordings. After each recording the entire cage was cleaned with ethanol before reusing.

### USV emission during same- and opposite-sex encounters

We recorded 20 male and 20 female mice emitting vocalizations in response to same- and opposite-sex stimuli (n = 40). Subjects were always primed with an individual of the same sex as the stimulus used for the recording session (e.g., when a male subject was recorded with a female stimulus, it was primed with a female, whereas when a male was recorded with a male stimulus, it was primed with a male). The mice were divided into four different treatment groups (n = 10 mice each): (1) male focal subjects presented with female stimuli (M(f)); (2) male focal subjects presented with male stimuli (M(m)); (3) female focal subjects with male stimuli (F(m)); and (4) female focal subjects with female stimuli (F(f)). Note that the focal subjects are depicted with an uppercase letter (M or F), whereas the stimulus mice are represented with a lowercase letter (m or f).

### Automatic USV data processing

To process our sound files we implemented the Automatic Mouse Ultrasound Detector (A-MUD 1.0), which applies a segmentation algorithm in a new script in STx (S_TOOLS-STx version 4.2.2; Acoustic Research Institute, Vienna). The development and evaluation of A-MUD’s performance has been described elsewhere [53]. In short, A-MUD automatically detects USVs in the sound file and provides a spectrographic analysis for each detected element (i.e., candidate syllable) and the ability to quantify frequency, amplitude and time parameters. A-MUD 1.0 has a detection threshold to reduce error rates and it does not detect very short calls (<10 ms), which are difficult to distinguish from background noise [53]. A-MUD’s error rates were evaluated under the same conditions as in this study and we found more correct positives, fewer false positive and fewer false negative elements than a commercial software used as comparison [53].

### Statistical analyses

Results are reported as mean ±1 standard deviation, and statistical analyses were conducted in IBM SPSS Statistics 22. To analyze amount and rate of USV emission, we used the total number of elements detected per file during the 10 min recording (‘number of elements’). To analyze spectrographic features (i.e., frequency and amplitude parameter information), we used the mean of the elements detected per file to calculate a grand mean per file, separately for each parameter (i.e., mean of minimum frequency, mean of maximum amplitude, etc.; see below). To investigate group differences in number of elements, we calculated generalized linear models (GZLM) with a negative binomial log link function model type, as recommended for count data [54], and robust estimators for the covariance matrix to handle violations of the model assumptions (unless stated otherwise). For analyzing spectrographic features of USVs, we first conducted principal component analyses (PCA) for all frequency and amplitude parameters. These analyses showed that all frequency parameters (mean of mean frequency, mean of minimum frequency, mean of maximum frequency and mean of frequency at the point of maximum amplitude) could be combined into one component (PC 1), which explained 94% of the total variance. The amplitude parameters (mean of mean amplitude, mean of maximum amplitude, mean of amplitude at the point of minimum frequency and mean of amplitude at the point of maximum frequency) could also be combined into one component (PC 1), which explained 75% of the total variance. Given that all frequency or amplitude parameters were highly correlated, we used only the mean of the mean frequency and mean of the mean amplitude of the elements for further analysis. To investigate group differences in grand mean frequency, we calculated GZLM in a linear model type and used robust estimators for the covariance matrix and to investigate differences in grand mean amplitude, we utilized general linear models (GLM) (unless stated otherwise). We performed non-parametric statistics when the assumptions of parametric statistics were not met, used two-tailed tests, and results are considered statistically significant at α≤0.05.

### Ethical statement

This study was carried out in strict accordance with the recommendations in the Guide for the Care and Use of Laboratory Animals of the National Institutes of Health. All the experiments were conducted at the Konrad Lorenz Institute of Ethology, Austria and the protocols have been approved and were in accordance with ethical standards and guidelines in the care and use of experimental animals of the Ethical and Animal Welfare Commission of the University of Veterinary Medicine, Vienna (Austria). We did not sacrifice any of the mice used for this study.

## Results

**Fig 1:**
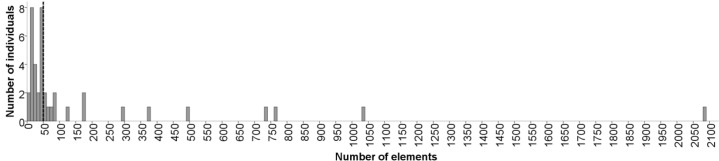
Histogram showing variation in the vocal behavior (number of calls) among individuals. Many mice produced few calls (≤50 elements) during the 10 min trials, though some mice were highly vocal (n = 40). The vertical dashed line shows the arbitrary cutoff used to distinguish between the low and high callers.

As expected, we found high individual variation in the number of elements emitted during the 10 min trials, and some mice produced few, if any vocalizations (rate: 4 to 2083 elements/10 min trial). There was a positive and significant skew in the distribution of the number of elements emitted (skewness = 3.7; kurtosis = 15.5, K-S = 0.35, p<0.001; Fig 1). Visual inspection of the data suggests that a slight majority (60%) of mice could be classified as ‘low callers’ that emitted few calls (≤50 elements), whereas fewer (40%) could be considered ‘high callers’ (51 to 2083 elements). This distinction between high versus low callers was arbitrary and made merely for exploratory purposes.

**Fig 2:**
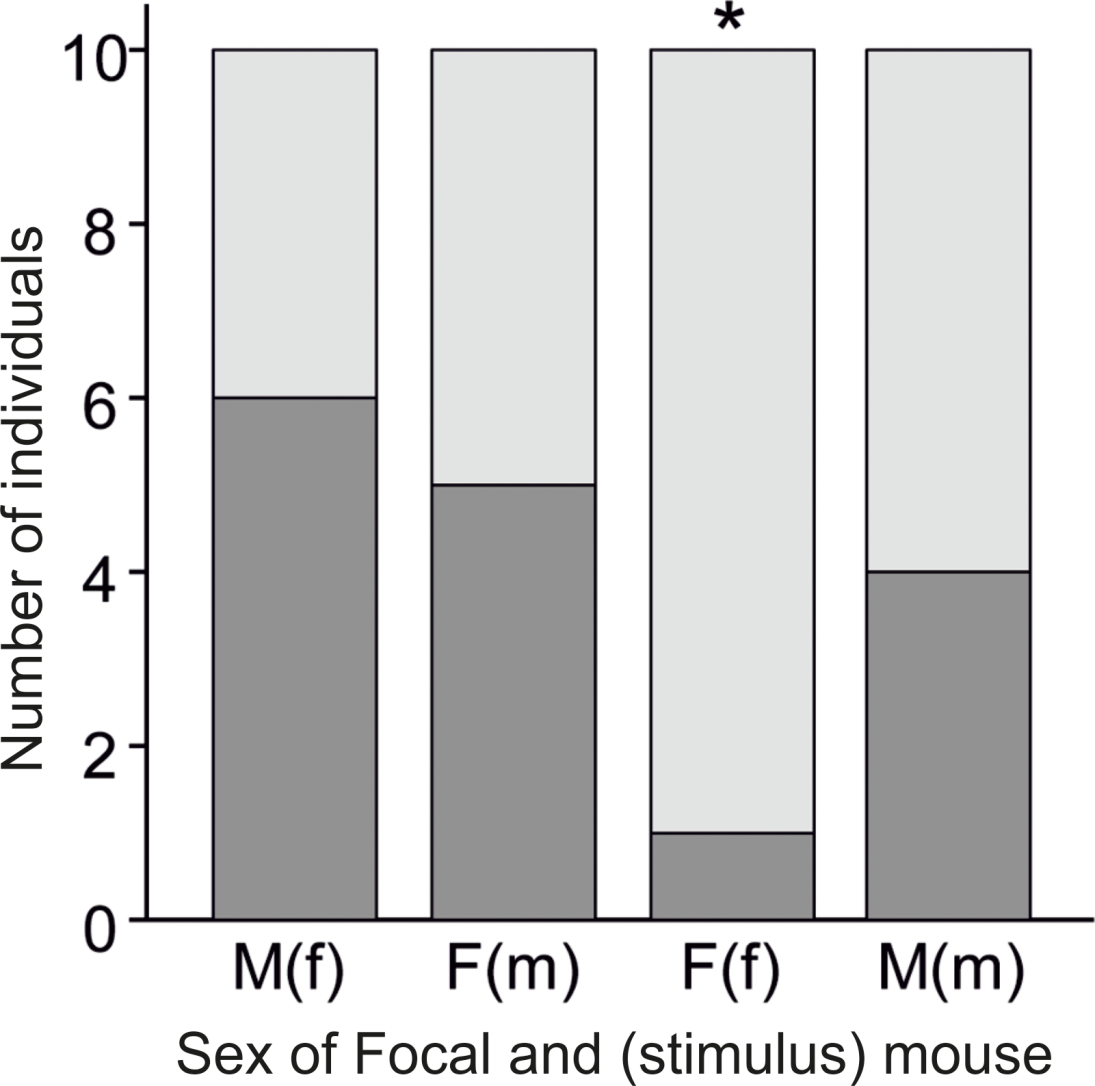
Number of subjects that were high or low callers when presented with individuals of the same- or opposite-sex. Number of individuals that emitted > 50 elements (‘high callers’: dark gray) and ≤50 elements (‘low callers’: light gray) during 10 min recordings of males (M) and females (F) in the presence of a female (f) or male (m) stimulus (n = 10 per group). The sex of focal animal is depicted in capital letters and sex of the stimulus animal in brackets. * = p<0.05.

Since most mice produced few vocalizations (Fig 1), we first conducted simple exploratory analyses to test whether the sex of the subject (caller) or stimulus explained the skewed distribution of vocalizing mice. We found that 10/20 males were high callers, and 6/20 females were high callers, regardless of the stimulus sex (Fig 2). We also found that 11/20 mice were high callers when tested with the opposite sex, and 5/20 mice were high callers when tested with the same sex (χ^2^ = 3.75, p = 0.053). This trend can be explained by a low number of high callers in trials with same- (Binomial test: 50% probability, p = 0.04) versus opposite-sex stimuli (Binomial test: 50% probability, p = 0.82). When investigating each of the combinations separately, we found significantly fewer high callers (1/10) when females were recorded in the presence of female stimuli (Binomial test: 50% probability, n = 10, p = 0.0215, Fig 2).

**Fig 3:**
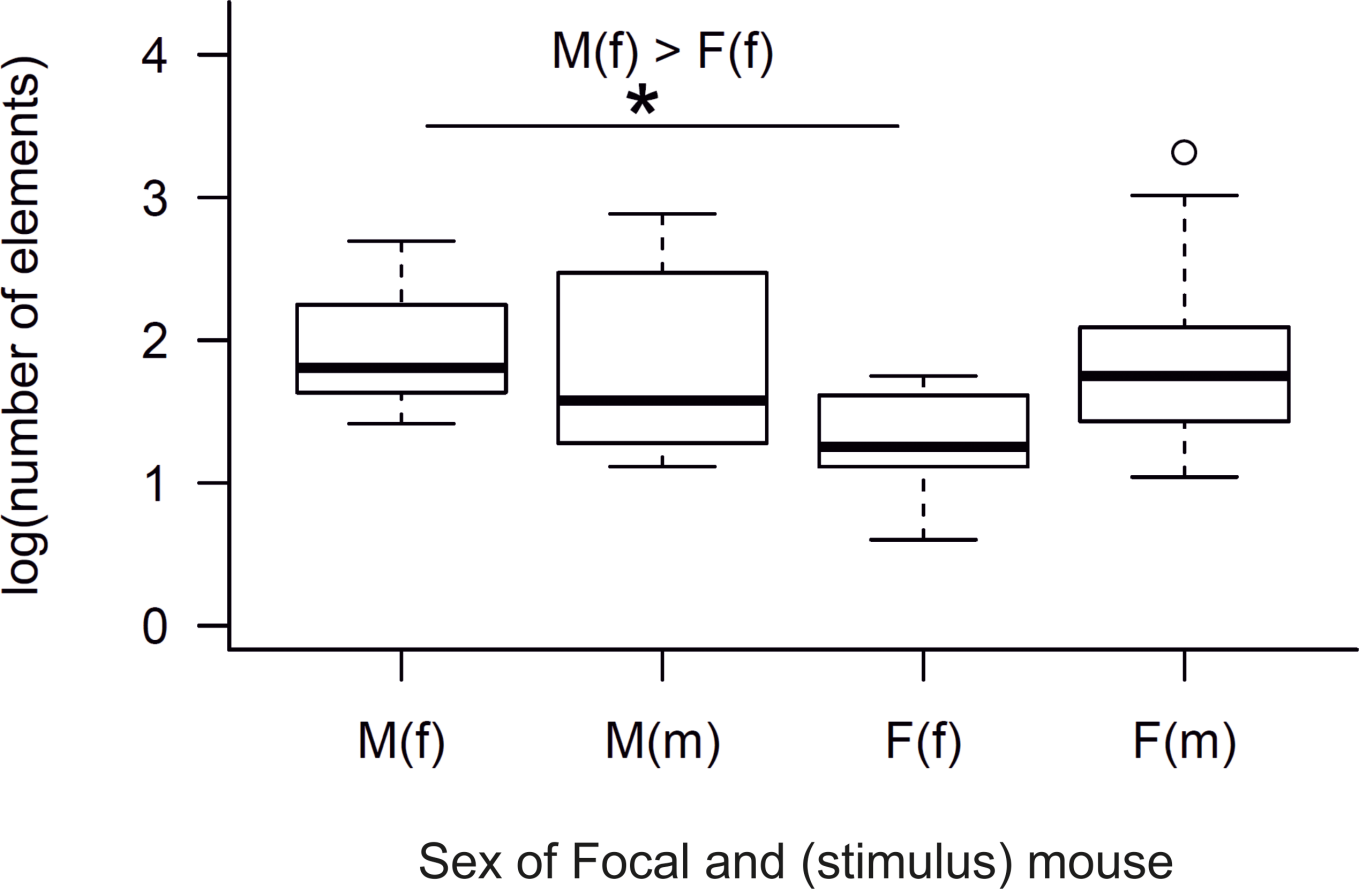
Number of USV elements emitted when subjects were presented with same- or opposite-sex individuals. Boxplots of number of elements (logarithmic scale) emitted by the focal mice in the presence of the stimulus mouse (n = 10 per group). The sex of focal animal is depicted in capital letters and sex of the stimulus animal in brackets. The graph shows median ± 95% CI, including the 25th and the 75th percentiles. * = p<0.05. ^o^ = outlier data point laying outside the whiskers of the boxplot.

For a more rigourous comparison, we tested whether the total number of elements emitted by individual mice depended upon the sex of the caller, the receiver, or both, and found a significant difference in the number of elements among the four possible interactions (Kruskal-Wallis H test: n = 40, χ^2^(3) = 8.544, p = 0.036, with a mean rank score of 25.85 for M(f), 23.40 for F(m), 11.60 for F(f) and 21.15 for M(m), Fig 3). Post-hoc analysis with Dunn-Bonferroni pairwise comparisons showed that there was a significant difference between males presented with females versus females presented with females (Z = 14.25, p = 0.038). When investigating whether the number of elements depends upon the sex of the caller mouse, the sex of the stimulus mouse or their interaction, we found that the sex of the stimulus mouse and the interaction between the sex of the caller and stimulus mouse had a significant effect in the model, but not the sex of the caller mouse (GZLM, n = 40; sex of stimulus: Wald-χ^2^(1) = 13.46, p<0.001, sex of caller: Wald-χ^2^(1) = 2.11, p = 0.146, sex of caller * sex of stimulus: Wald-χ^2^(1) = 7.27, p = 0.007). We also investigated whether the number of elements depended on whether the mice were vocalizing for same or opposite sex, independently of the sex of callers or stimulus and found that opposite-sex groups evoked significantly more elements (mean rank = 24.63) than same-sex groups (mean rank = 16.38), (Mann-Whitney test: n = 40, Z = - 2.233, p = 0.026).

**Fig 4:**
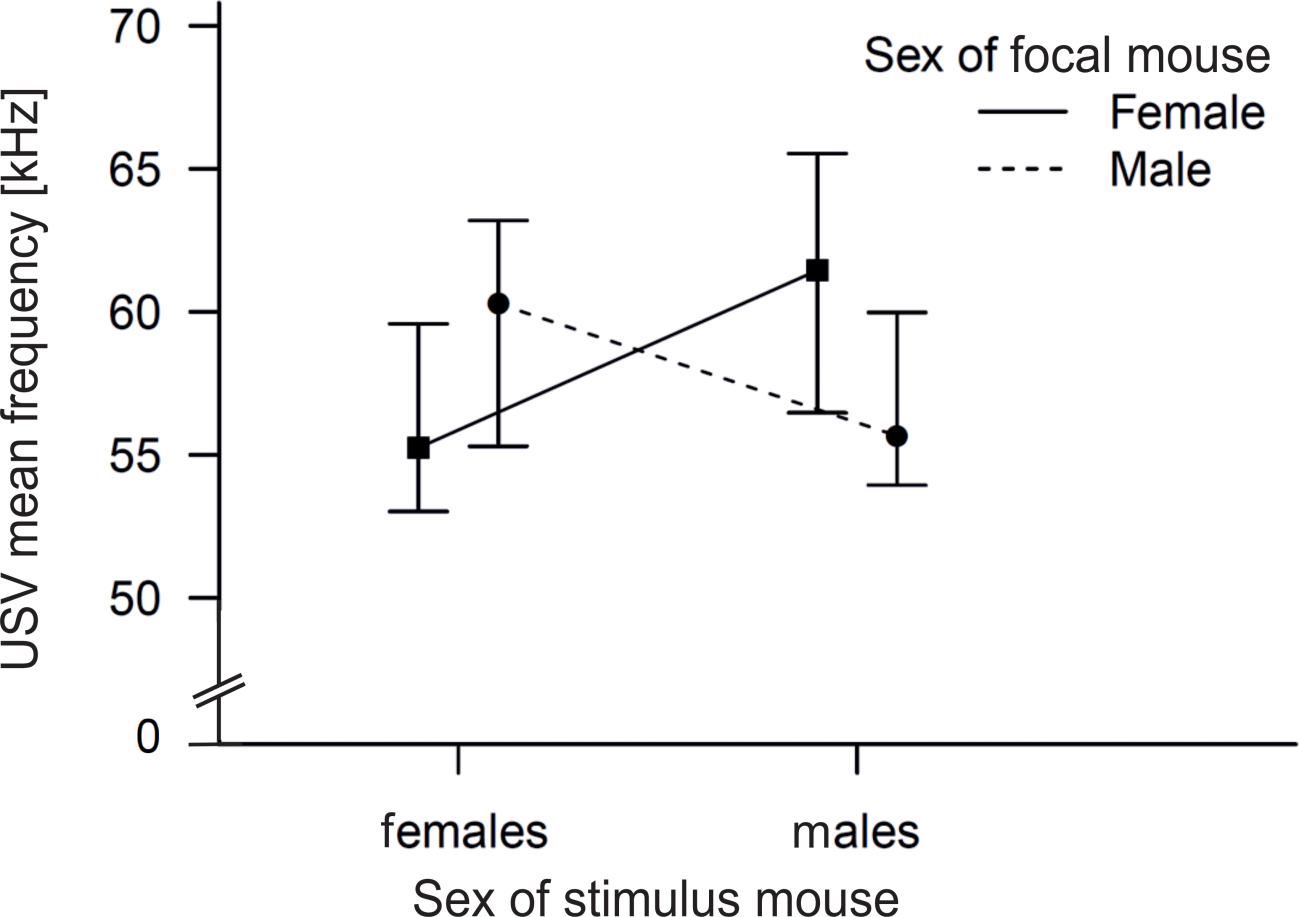
Frequency of USVs emitted when subjects were presented with same- or opposite-sex individuals. Mean USV frequency of elements emitted by the focal to the stimulus mouse, depicted by sex (n = 10 per group). The figure shows median ± 95% CI.

We found no difference in the frequency of the vocalizations emitted by the individual mice among the four groups (Kruskal-Wallis H test: n = 40, χ^2^(3) = 4.4, p = 0.221). We also tested whether the mean frequency was dependen**t** on the sex of the caller, the sex of the stimulus or their interaction and we found that neither the sex of the stimulus, nor the sex of the caller mouse were significant in the model, however, the interaction was significant (GZLM, n = 40; sex of stimulus: Wald-χ^2^(1) = 0.59, p<0.442, sex of caller: Wald-χ^2^(1) = 0.122, p = 0.727, sex of caller * sex of stimulus: Wald-χ^2^(1) = 4.964, p = 0.026, Fig 4). Further, we investigated whether the mean frequency depended on whether the mice were vocalizing for the same or opposite sex, independently of the sex of callers or stimulus and found that opposite sex groups evoked significantly higher mean frequency (mean rank = 24.20) than same sex groups (mean rank = 16.80), (Mann-Whitney test: n = 40, Z = - 2.002, p = 0.045). The mean frequency ranged from 52.17 kHz to 69.17 kHz (mean±SD = 59.25±5.51 kHz) when males vocalized in the presence of a female stimulus and from 51.22 kHz to 69.81 kHz (mean±SD = 61.00±6.33 kHz) when females vocalized for a male stimulus. On the other hand, in same-sex encounters, males emitted USVs with a mean frequency from 52.24 kHz to 64.57 kHz (mean±SD = 56.96±4.22 kHz) and females from 51.22 kHz to 64.01 kHz (mean±SD = 56.30±4.59 kHz).

We also examined the mean amplitude of USVs emitted by the mice, and found no difference between the four groups (GLM: n = 40, F = 1.951, df = 3, p = 0.139). When analysing only male callers, however, we found a trend towards higher amplitude, when males were exposed to male (mean±SD = -83.22±2.9 db) compared to female stimuli (mean±SD = -85.32±1.2 db; T-test for unequal variances: n = 20, t = -2.108, df = 12.001, p = 0.057). We tested whether the mean amplitude depended upon the sex of the caller, the sex of the stimulus or their interaction, and found that neither the sex of the stimulus, the sex of the caller, nor their interaction were significant in the model (GLM, n = 40; sex of stimulus: F = 2.77, df = 1, p = 0.105, sex of caller: F = 1.538, df = 1, p = 0.223, sex of caller * sex of stimulus: F = 1.544, df = 1, p = 0.222). We then investigated whether the mean amplitude depended on whether the mice were vocalizing for same or opposite sex, independently of the sex of callers or stimuli and, again, found no differences (T-test for unequal variances: n = 40, t = -1.207, df = 33.215, p = 0.236) (S1 Table).

## Discussion

We found that USV emission rates were highly variable among individuals (4 to 2083 elements/10 min trial), and most (60%) mice emitted few (≤50) calls. The proportion of mice that vocalized at high rates (>50 calls) depended upon the sex of caller and the stimulus mouse, i.e., there was a higher proportion of ‘low callers’ in trials with the same- versus opposite -sex stimuli, and in particular, there were significantly more low callers among females presented with another female (9/10) compared to the other combinations (Fig 2). Our subsequent analyses of the total number of USVs were consistent with these first exploratory analyses, and indicated that (a) both sexes emitted more USVs when presented with opposite- than same-sex individuals; (b) mice modulated the rate and frequency of their USVs depending upon the sex of the stimulus; and (c) females vocalized at lower rates than males in response to a female stimulus (Fig 3). We also detected a trend in the recorded amplitude, such that the amplitude tended to be higher when males called for other males than for females. This trend in amplitude variation may be due to males producing louder vocalizations, or changing their distance from the microphone, or their orientation, or some combination of these explanations. These are all non-mutually exclusive mechanisms that can potentially influence amplitude, though changes in amplitude perceived by the receiver might be the same regardless of the underlying mechanism. Taken together, our results indicate that there is high individual variation in USV emission when mice are presented with a stimulus individual, and that individual USV emission depends upon the sex of the caller and the receiver.

Our results are consistent with a previous study that found that mice emit more USVs during opposite- than same-sex interactions (though statistical analyses were not reported) [34]; but differ from recent studies reporting that the highest rates of calling were found during female-female interactions (laboratory mice [50]; wild-derived *Mus musculus domesticus* [51]). Our study was conducted with *Mus musculus musculus*, and the low rates calls that we observed during female-female interactions could be due to differences in the vocal behavior of these subspecies. *M*. *m*. *musculus* females may be more aggressive and less social than *M*. *m*. *domesticus*, and do not appear to engage in cooperative breeding (SZ, DR, DP person. obser.). The disparities might also be due to differences in methods and contexts of recording. For example, one study recorded USVs during 3-min intruder tests [50], and the other analyzed the first 30 ‘songs’ emitted during a process of familiarization through a divider over two days and two nights [51]. In contrast, we recorded mice during 10-min encounters in response to an unfamiliar individual separated by a divider–after the mice were primed with an individual of the same sex as the stimulus mouse (social experience). Our findings might also be explained by sex differences in USV emission in response to the sex used for social priming (e.g., female priming may reduce female but not male calling for unfamiliar females). This hypothesis is testable. Interestingly, an early study found that female USV emission changes over time depending upon the sex of the stimulus individual (i.e., when presented with an anesthetized mouse over several days, females initially emitted more USVs for females than males, but after 4 d, they reversed this pattern) [46]. Moreover, a recent study found that USVs recorded during opposite-sex interactions (assumed to be emitted mainly by males) become more complex over time, during the process of courtship and mounting [35]. It has been suggested that the brief 3 to 5 min trials usually conducted may not be sufficient for assessing USV emission, and it may only reflect the state of arousal during the initial stages of social interactions [49]. On the other hand, USVs emitted during initial contact may be critical for determining subsequent interactions. Future studies are therefore needed to examine how same- and opposite-sex social experience affects the USVs of males and females, how USVs change over time during repeated encounters, and how changes in USVs affect subsequent same- and opposite-sex interactions. Another source of variation in calling may have been the estrous state of the caller, as estrous females in one study emitted fewer USVs in response to a female intruder compared to non-estrous females [55]. Therefore, future studies should examine possible estrous effects when comparing male and females USV behavior.

Our study was limited to sex differences in vocalization rates or performance, and more work is needed to examine call structure and reportoire, especially by recording both individuals during direct interactions [30]. If USVs of both sexes are found to have similar structure and performance, however, this would not refute the courtship hypothesis, contrary to what it has been suggested, because traits need not be sexually dimorphic to provide courtship functions or to evolve under sexual selection [56]. Also, playback studies are needed to examine whether modulating vocalizations to higher frequencies alters how mice are pereceived when assessing a potential mate or competitor. Studies with other species have examined whether male vocalizations are honest or dishonest (exaggerated) indicators of size [57–59]. Voice preference studies with human subjects have found that men preferred women with a higher pitched voice [60, 61], and that lower pitched voices are perceived as more dominant [60, 62]. We are unaware of any evidence that mouse USVs convey honest (or exaggerated) signals of size [8].

We found highly variable rates of USV emission among individual mice, and though the rate of calling was low (overall 14 elements/min on average), it was nearly identical to a previous study conducted on F1 wild-caught mice from the same population in our laboratory (13 elements/min) [21]. In the previous study, however, 20% of males did not vocalize during the 90 min recording sessions, and were excluded from the analysis [21]. Non-vocalizing in the previous study might be explained by the stimulus provided (mouse urine rather than individual mice), though a study with a domesticated strain found no differences in the number of USVs males emitted to these types of stimuli [11]. The proportion of males that vocalize or not in response to a female interaction varies among mouse strains [34]. There are also differences in USVs among *Mus musculus* populations and between *Mus* species [26, 51]. USV emission of wild mice is much lower than domesticated mice (e.g., male laboratory mice emit 90 elements/min on average in responses to fresh female urine [63]), and up to 160 elements/min in response to anesthetized mice [50], though standardized comparisons have not been conducted. Future studies are needed to determine why there is so much individual variation in USV emission, and why some mice emit few or any USVs (USV studies commonly screen mice and only investigate highly vocal individuals).

To conclude, our results provide one of the first comparisons of the vocalizations between the sexes in wild-derived house mice, and the first study with *Mus musculus musculus*. Our results differed from previous studies that recorded rates of USV emission in female-female encounters (wild-derived *Mus musculus domesticus* [51]; laboratory mice [14]). Further studies would be useful to record USV emission of wild mice in natural or naturalistic housing conditions, and playback experiments are needed to determine the consequences of modulating USV emission in response to encounters with the same or opposite sex.

## Supporting information

S1 Table Data: Total number of elements, frequencies and amplitudes of emitted USVs. (XLSX)
